# The Presence of Two Distinct Lineages of the Foot-And-Mouth Disease Virus Type A in Russia in 2013–2014 Has Significant Implications for the Epidemiology of the Virus in the Region

**DOI:** 10.3390/v17010008

**Published:** 2024-12-25

**Authors:** Victor V. Nikiforov, Sergey A. Noskov, Alexander V. Sprygin, Mohammad Abed Alhussen, Anastasia S. Krylova, Taisia V. Erofeeva, Svetlana N. Fomina, Svetlana R. Kremenchugskaya, Fedor I. Korennoy, Maxim V. Patrushev, Ilya A. Chvala, Tamara K. Mayorova, Stepan V. Toshchakov

**Affiliations:** 1 Federal Center for Animal Health FGBI ARRIAH, 600901 Vladimir, Russia; sprygin@arriah.ru (A.V.S.); alhussenmohammed85@hotmail.com (M.A.A.); fomina@arriah.ru (S.N.F.); kremenchugskaya@arriah.ru (S.R.K.); korennoy@arriah.ru (F.I.K.); chvala@arriah.ru (I.A.C.); mayorova@arriah.ru (T.K.M.); 2 National Research Center “Kurchatov Institute”, 123182 Moscow, Russia; noskov_sa@nrcki.ru (S.A.N.); krylova.gen@gmail.com (A.S.K.); erofeeva_tv@rrcki.ru (T.V.E.); patrushev_mv@nrcki.ru (M.V.P.)

**Keywords:** foot-and-mouth disease, serotype A, SEA97, Iran-05, FMDV outbreak, Russia, molecular epidemiology, phylogeny, vaccine matching

## Abstract

Molecular surveillance of FMD epidemiology is a fundamental tool for advancing our understanding of virus biology, monitoring virus evolution, and guiding vaccine design. The accessibility of genetic data will facilitate a more comprehensive delineation of FMDV phylogeny on a global scale. In this study, we investigated the FMDV strains circulating in Russia during the 2013–2014 period in geographically distant regions utilizing whole genome sequencing followed by maximum-likelihood phylogenetic reconstruction of whole genome and VP1 gene sequences. Phylogenetic analysis showed congruence in the topology of the phylogenetic trees constructed using the complete genome and VP1 gene sequence, clearly demonstrating that the isolates analyzed belong to two distinct genetic lineages: A/SEA97 in the Far East and Iran-05 in the North Caucasus. The A/SEA97 isolates exhibited a close genetic identity to those from China and Mongolia, whereas the Iran-05 isolates demonstrated clusterization with those from Turkey. The vaccine-matching studies with isolates from the Far East and North Caucasus revealed no antigenic homology with A/SEA-97 (r_1_ = 0.015–0.29) and A/Iran 05 (r_1_ = 0.009–0.17). The close genetic relationship of FMDV in the reported outbreak waves to those from neighboring countries indicates that animal movement could contribute to spillover and virus dispersal. The phylogenetic data reported here provide insight into the molecular epidemiology of FMD in the Eurasia region, elucidating the circulation pattern, molecular evolution, and genetic diversity, which is highly valuable for guiding vaccine designs and improving regional eradication policies.

## 1. Introduction

The foot-and-mouth disease virus (FMDV) is a member of the Picornaviridae family, within the genus Aphthovirus [1]. The susceptible host range includes buffaloes, cattle, goats, sheep, and pigs [2]. The FMD genome is a single-stranded positive-sense RNA of 8500 base pairs in length. The viral capsid includes the structural proteins VP1, VP2, VP3, and VP4, while non-structural proteins are mainly involved in viral replication and pathogenesis [3]. The VP1 protein is the primary immunogenic component of the virion and serves as the principal phylogenetic target for genome-based epidemiology of the virus [4].

The primary means of transmission of the virus is via alimentary and airborne routes, as evidenced by the occurrence of infected or reconvalescent animals when they share pastures with wild animals. The bodily secretions and excretions of infected animals, including saliva, nasal discharge, milk, semen, and even exhaled air, serve as a source of infection, infecting other animals in contact with the source and contaminating feed, litter, and water troughs. The pathogen has the capacity to disseminate over considerable distances via a variety of vectors, including wind and fomites such as staff clothing and footwear, vehicles, and other materials [5].

Given the error-prone replication and high evolutionary rates of mutation, foot-and-mouth disease virus (FMDV) exists as seven immunological serotypes, distinguished by differences in the nucleotide sequences of the VP1 protein. The seven immunological serotypes of FMDV, designated A, O, C, Asia-1, SAT-1, SAT-2, and SAT-3, exhibit a high degree of diversity, encompassing a multitude of topotypes, genetic lineages, and strains [6]. The distribution of FMD serotypes in endemic regions is uneven, which makes the FMD molecular epidemiology a crucial element of disease study and control programs [7]. It should be noted that there is no cross-protection between different serotypes. Consequently, infection or vaccination with one FMD serotype does not provide protection against infection with other serotypes of the pathogen [8].

A review of the most recent data from the World Organization for Animal Health (WOAH) and media reports suggests that, despite the implementation of control measures, the epizootic situation of foot-and-mouth disease (FMD) remains a significant global concern. As indicated by official data, the disease affected 65 countries between 2000 and 2022. Of these, 25 were in Asia, 37 in Africa, 2 in Europe, and 1 in South America. Furthermore, five known types of FMD were identified: type O in 44 countries, type A in 25, type SAT-1 in 10, type SAT-2 in 14, and type Asia-1 in 5. However, the pathogen type was not determined in 14 African countries. In some countries, the circulation of two to four FMDV types has been documented (Afghanistan, Vietnam, the Democratic Republic of the Congo, Egypt, Iran, Kenya, China, Thailand, Tanzania, Turkey, etc.). The active circulation of diverse virus strains and the occurrence of successive waves of infection serve to accelerate the spread of the virus in FMD-free regions. In this context, genomic analysis tools are of particular importance for analyzing the molecular aspects of FMD virus evolution and spread.

Serotype A is classified into three topotypic profiles: Asia, Europe–South America (Euro-SA), and Africa. The Asia topotype is the most prevalent in the Middle East and South Asian and West Eurasian countries [9]. In its turn, each topotype is further subdivided into genetic lineages, named after the country where and when it was recovered, for example, A-IRN99, A-Iran05, A-IRQ24,46, and A-TUR-2006 [10]. Egypt is affected by the African topotype [11].

Russia borders on countries with a history of FMD circulation, which heightens risks of FMDV transboundary incursion into the country [12,13,14]. A total of 94 outbreaks of foot-and-mouth disease (FMD) have been reported in Russia between 2005 and 2024. However, until 2013, the cases registered in the Russian Federation were of serotype O, and serotype A had not been detected for more than 20 years [15]. In 2013, however, the situation became dramatically more challenging, with 21 outbreaks of FMD type A detected in the Far Eastern, Siberian, and North Caucasian Federal Districts. In particular, nine and six outbreaks were identified in 2013 in Zabaikalsky Krai and Amur Oblast, respectively, with the epizootic continuing in 2014–2015. Additionally, two outbreaks were identified in Karachay-Cherkessia, three in Krasnodar Krai, and one in Kabardino-Balkaria, marking the first occurrence of such incidents in decades in the North Caucasus region. Outbreaks of this serotype of FMD have also been reported in Mongolia and eastern Kazakhstan [16]. Since 2013, Russia has had a buffer zone along its southern borders where prophylactic vaccination of cattle and livestock with trivalent FMD vaccine types A, O, and Asia-1 has been carried out twice a year. Currently, according to WOAH, more than 50 regions of Russia have the status of FMDV-free zones without vaccination, and four zones along the southern borders (South, East Siberia, Far East, and Ural-West Siberia) have the status of FMDV-free zones with vaccination (https://www.woah.org/app/uploads/2023/05/russia-eng.png) (accessed on 20 May 2023).

In the 2013–2014 period, among the FMDV outbreaks affecting pigs, cattle, and small ruminants, 28% were attributed to serotype A. However, no detailed molecular epidemiologic studies have been conducted to elucidate the circulation pattern of this serotype [17]. The present study reports the detection and characterization of the molecular epidemiology of the FMDV type A in Russia during 2013–2014, employing a range of methods including serological, antigenic, and phylogenomic analyses.

## 2. Materials and Methods

### 2.1. Sampling and FMD Data

This study was conducted using aphthous epithelium (vesicle walls) collected from cattle suspected of being infected with FMD. The biological samples were submitted to the FGBI “ARRIAH” (OIE Regional Reference Laboratory and FAO Reference Center for FMD) from the Far East and North Caucasus regions of the Russian Federation during the period 2013–2014.

The data on FMD outbreaks in the Russian Federation for the period 2013–2014 were retrieved from the WOAH World Animal Health Information System (https://wahis.woah.org/#/event-management, accessed on 19 December 2024). In this study, an outbreak was defined as a geographically distinct occurrence of laboratory-confirmed FMD in one or several animals officially reported to the WOAH. Laboratory confirmation was conducted by the Federal Center for Animal Health (FGBI “ARRIAH”).

### 2.2. Virus Isolation

For the isolation and propagation of the FMDV, continuous monolayer cell cultures of Siberian ibex kidney cells (PSGK-30) and pig kidney cells from Instituto Biologico-Rim Suino-2 (IB-RS-2) were utilized. The infected cell cultures (CCSs) were incubated at 37 °C until the full cytopathic effect (CPE) was observed. A maximum of three passages were performed in the cell culture for the purpose of virus adaptation. The virus was deemed to have been adapted if it resulted in 90–100% CPE within 18–24 h (https://www.woah.org/fileadmin/Home/eng/Health_standards/tahm/3.01.08_FMD.pdf, accessed on 19 December 2024).

Viral infectivity titration by the micromethod was conducted in 96-well culture plates on a continuous IB-RS-2 culture of cells with a concentration of 0.8–1.0 × 10^6^ cells/mL at 37 °C and 5% CO_2_ for 48 h. The viral titer was determined by observing the number of wells exhibiting a characteristic CPE under an inverted microscope, as described by Karber, and expressed as log TCD50/mL (TCD, tissue cytopathic dose). This procedure was conducted at 37 °C and 5% CO_2_ for 48 h.

### 2.3. Antigenic Matching of FMD Virus Isolates

The reference sera were provided by the FGBI “ARRIAH” from cattle vaccinated with FMD monovalent inactivated vaccines from the following type A strains at 21–30 days post-vaccination: A22 No. 550/Azerbaijan/64, A22/Iraq/64, A/Iran/97, A No. 2029/Turkey/06, A No. 2045/Kyrgyzstan/07, A No. 2155/Zabaikalsky/2013, and A No. 2166/Krasnodarsky/2013.

FMD viral isolates were matched with the production strain in the microneutralization (MN) reaction in the IB-RS-2 cell culture. The titers of reference sera of cattle vaccinated with FMD vaccines developed from homologous and heterologous FMD strains were determined by the checkerboard method using five doses of virus. The serum titer against 100 TCD_50_ was calculated using linear regression and expressed as log.

The r_1_-value was defined as the reciprocal arithmetic log10 titer of reference serum against both heterologous and homologous viruses. The value was interpreted in accordance with the methodology proposed by M. Rweyemamu (1984), whereby an r_1_ value of ≥0.3 indicates a close antigenic relationship among the strains; therefore, the use of a vaccine based on this production strain is likely to confer protection against challenges with the field isolate [18]. Conversely, r_1_ < 0.3 indicates no antigenic relationship among the strains, and the production strain does not confer protection against the field isolate. The cut-off value range of r_1_ was 0.28–0.32.

### 2.4. Sequencing of FMDV Type A Strains

RNA was extracted from cell culture supernatants using Trizol™ reagent (Thermo Fischer Scientific, Waltham, MA, USA) in accordance with the manufacturer’s instructions. Due to the significant contamination of RNA preparations with DNA, as revealed by fluorometry on the Qubit™ (Thermo Fischer Scientific, USA) with DNA- and RNA-specific dyes, samples were subjected to an additional round of treatment with RNase-Free DNase Set and repurified with RNeasy™ Mini Kit (both Qiagen, Germany), according to supplied protocols. The resulting RNA concentrations were in the range of 84–200 ng/μL.

RNAseq libraries were prepared from 100 to 500 ng of RNA using the NEBNext^®^ Ultra™ II RNA Library Prep Kit for Illumina (New England Biolabs, Ipswich, MA, USA) using a random primer-based protocol without prior enrichment. The library quality was assessed with the TapeStation 4200 automated electrophoresis system (Agilent Technologies, Santa Clara, CA, USA). The peak distribution maximum of the obtained libraries was in the range of 320–370 bp.

Sequencing was performed on a NovaSeq™ 6000 system (Illumina, San Diego, CA, USA) utilizing an SP flow cell with 2 × 100 bp paired-end reads. Seven libraries were sequenced in total, resulting in 9.06–23.02 million raw read pairs per library.

### 2.5. De Novo Assembly of FMDV Type A Strain Genomes

The raw reads were curated in three stages. Initially, the reads were filtered by quality using the positional quality over a flowcell with the *filtebytile.sh* script from the BBTools package [19]. Subsequently, the adapter sequences were removed, and the reads were trimmed by quality with the *fastp* package [20]. Finally, the read coverage was normalized to 120×, and low-covered contamination from the cell line RNA was removed with the *bbnorm* script [19].

*De novo* assembly was conducted with the SPAdes v. 3.15.0 assembler in the *rnaviral* mode [21]. The assembled viral contigs were then manually curated in CLC Genomics Workbench v. 24.0.1 (Qiagen, Germany) to ensure the absence of misassemblies. The final consensus was obtained by mapping the filtered and trimmed, but not normalized, sequencing reads to the viral contigs and extracting the consensus. Viral genomes were annotated using the VADR annotation suite, with RefSeq and high-quality, complete FMDV genomes employed as reference models [22].

### 2.6. Reconstruction of the Phylogenetic Trees

The genome dataset utilized for the reconstruction of the whole genome phylogenetic tree consisted of seven genomes obtained in the present study, complete FMDV serotype A genomes from the World Reference Laboratory for Foot-and-Mouth Disease (WRLFMD) database (https://www.wrlfmd.org/fmdv-genome/fmd-prototype-strains, accessed on 19 December 2024), and complete representative genomes of FMDV type A outbreaks. In total, 52 genomes were used for the alignment (see Supplementary Table S1).

For the phylogenetic tree on the VP1 gene, the sequences of this gene were extracted from the full-genome sequences prepared above, and 29 VP1 sequences from the WRLFMD database were incorporated into the resulting dataset. Thus, 79 sequences were utilized for VP1 gene alignment (Supplementary Table S2).

Sequence alignment was conducted using the MAFFT v7.520 package with the default parameters [23]. Alignment trimming was performed using Trimal v1.4 with the following parameters: all columns with gaps of more than 20% of sequences or similarity scores below 0.001 were removed [24]. Final alignments consisted of 8010 and 639 columns for whole FMDV genomes and VP1 gene sequences, respectively.

To identify the most suitable nucleotide substitution model, the modeltest-ng package was employed using maximum likelihood tree reconstruction [25]. The analysis yielded the GTR + I + G4 model as the optimal choice for both the full-genome data and the VP1 gene data, with substantial support from the Bayesian Information Criterion (BIC).

A phylogenetic reconstruction was performed with the aid of the RAxML-NG package, with 1000 bootstrap iterations [26]. The resulting phylogenetic tree was subsequently visualized using the Interactive Tree of Life (iTOL) web server [27].

A comparative analysis of nucleotide identities and sequence differences was conducted using the “Create Pairwise Comparison” tool of CLC Genomics Workbench v.24.0.1 (Qiagen, Germany). This analysis employed the alignments utilized for the tree reconstructions.

## 3. Results

### 3.1. FMD Outbreaks in the Russian Federation in 2013–2014

**Figure 1:**
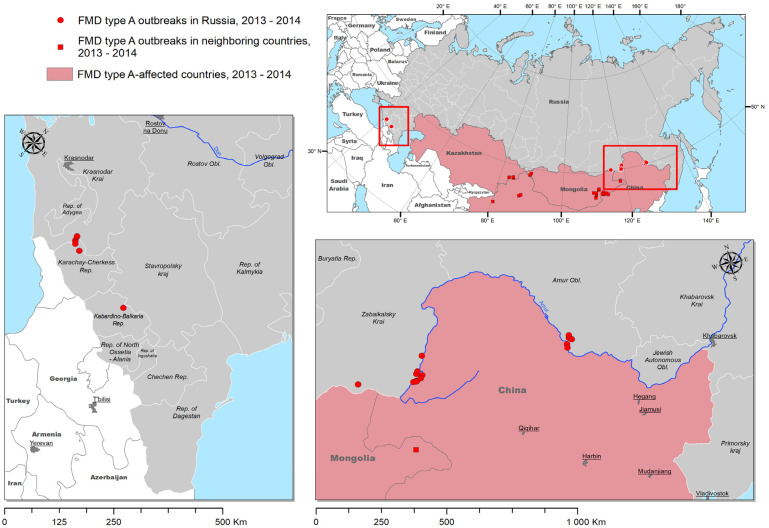
Map illustrating foot-and-mouth disease outbreaks in Russia over the period 2013–2014. Outbreaks of A serotype are shown as red circles; outbreaks of O serotype are shown as blue circles.

A total of 33 outbreaks were recorded in the Russian Federation over the period 2013–2014 in two regions, namely the North Caucasian and Far Eastern Federal Districts, situated in close proximity to the southern border of the country (Figure 1). Of the outbreaks, 24 (77%) were caused by FMDV serotype A, which had affected Russia for the first time in 2013. The remaining nine outbreaks were attributed to FMDV serotype O. All outbreaks of the O type emerged in Primorsky Krai in 2014, while the outbreaks of the A type affected both study areas in 2013 and 2014.

### 3.2. Virus Characterization

The main characteristics of the viral isolates described in this paper are summarized in Table 1.

Prior to molecular biological studies, viral infectivity titers were determined using the microtiter method in 96-well plates on IB-RS-2 culture cells and expressed as log TCD50/mL. The titration results of the FMDV type A isolates are presented in Table 2.

### 3.3. Viral Genome Reconstruction and Phylogenetic Analysis

A total of seven viral genomic libraries were sequenced on the Novaseq™ 6000 instrument (Illumina, San Diego, CA, USA), resulting in 9.06–23.02 million paired-end reads per sample. After de novo assembly of trimmed and normalized reads, seven full-genome viral contigs of length 8126–8259 bp were obtained. The coverage of viral contigs varied in the range of 3661×–35142× (Table 3).

Phylogenetic analysis demonstrated that FMDV strains isolated in the Russian Federation in 2013–2014 are part of the genetically diverse topotype Asia, which comprises multiple genetic sublineages. It is noteworthy that this topotype encompasses a genetically distinct line, SEA-97, which is genetically disparate from other representatives of the topotype. This observation may suggest the necessity for further reclassification of FMD virus genetic lines and topotypes.

**Figure 2:**
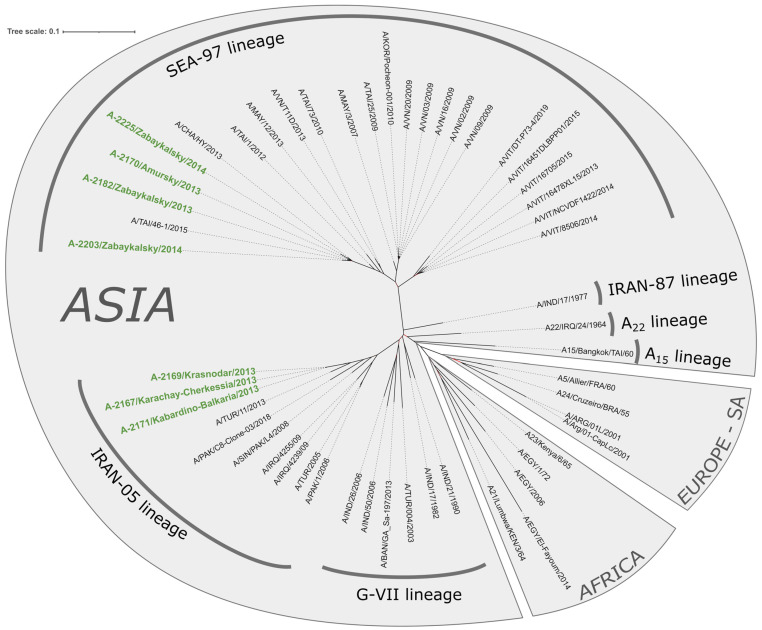
Maximum likelihood unrooted phylogenetic tree of the whole genome sequences of 52 serotype A FMDV isolates. The tree was constructed using RAXML-NG software with 1000 bootstraps [26]. The isolates described in this study are labeled with green. The nodes with bootstrap support less than 50% are marked with red. The topotype distribution is illustrated by light gray sectors. Genetic lineages within topotype ASIA (as referenced by https://www.wrlfmd.org/, accessed on 19 December 2024) are indicated by dark gray arcs.

**Figure 3:**
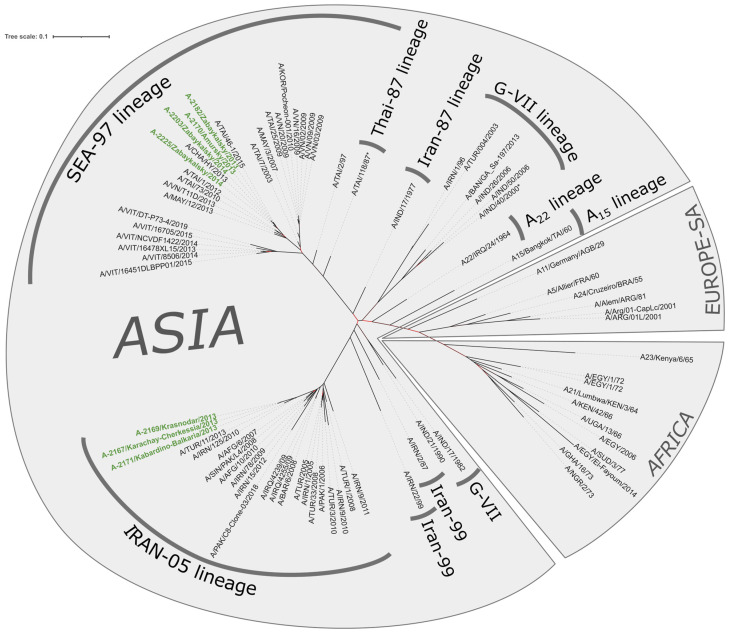
Maximum likelihood unrooted phylogenetic tree of the VP1 gene sequences of 79 FMDV isolates. The tree was constructed using RAXML-NG software with 1000 bootstraps [26]. The nodes with bootstrap support less than 50% are marked with red. The isolates described in this study are labeled with green. The topotype distribution is illustrated by light gray sectors. Genetic lineages within topotype A (as referenced by https://www.wrlfmd.org/, accessed on 19 December 2024) are indicated by dark gray arcs.

**Figure 4:**
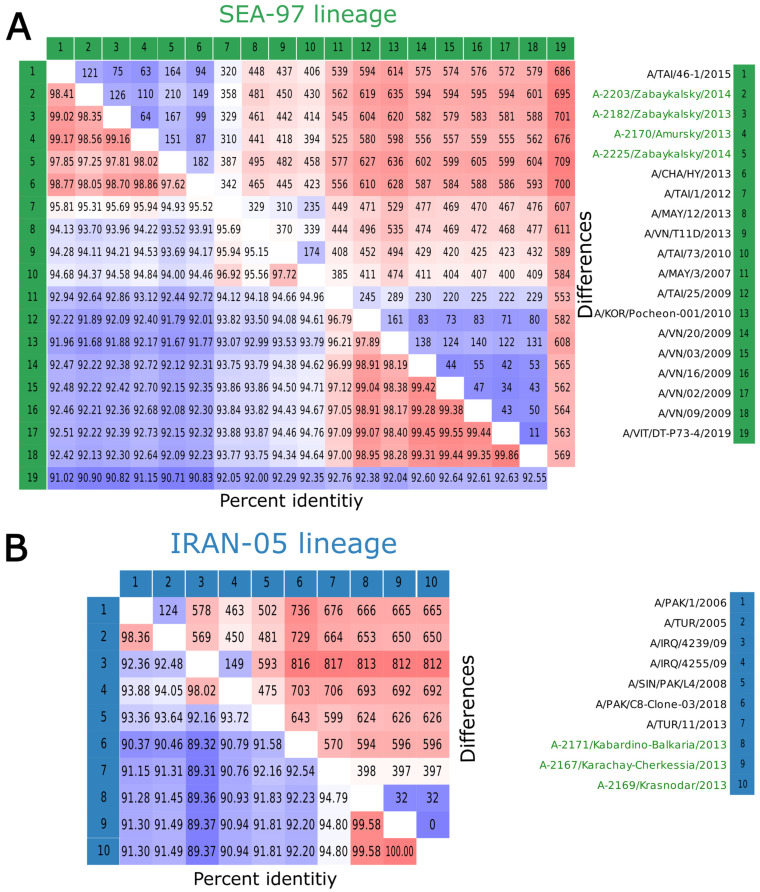
The pairwise comparisons of the whole genome FMDV serotype A sequences of SEA-97 (**A**) and Iran-05 (**B**) genetic lineages. The percentage of identical nucleotides is displayed in the bottom left half of each heatmap. The color scale varies from blue (minimum) to red (maximum). The top right half depicts the number of differences between the two sequences, including both gaps and single nucleotide polymorphisms (SNPs).

Isolates A-2167, A-2171, and A-2169 constitute a distinct and well-defined cluster within the Indo-Pakistani branch (Figure 2 and Figure 3), which has been designated as the genetic lineage Iran-05. The most closely related isolate from our dataset was identified as the FMDV strain A/TUR/11/2013, which was isolated in the Black Sea region of Turkey [28]. The pairwise analysis of nucleotide identities within the Iran-05 sublineage revealed that all analyzed strains exhibited 84.8% identities with Turkish strains, which displayed 397–398 differences (SNP and small indels) between their genomes. The Kabardino-Balkarian strain exhibited 32 polymorphisms from the A-2167 and A-2169 strains, which were almost identical within the cluster (zero differences in the trimmed alignment) (Figure 4B). A comparative genomic analysis of the VP1 dataset revealed the existence of a highly homologous strain, designated A/IRN/125/2010, that exhibited a nucleotide divergence of 22–25 nucleotides from the Russian strains, in addition to the previously identified A/TUR/11/2013 strain (Supplementary Figure S1).

Isolates A-2170, A-2182, A-2203, and A-2225 exhibited a close phylogenetic relationship with strains isolated in Thailand, Vietnam, Malaysia, and China between 2010 and 2015 (Figure 2 and Figure 3). These strains belong to the diverse SEA-97 (South-East Asia) genetic lineage [29,30]. Pairwise comparisons demonstrated that the Far East isolates formed a distinctive cluster, exhibiting a high level of similarity to the Chinese A/CHA/HY/2013 and Thai (A/TAI/46-1/2015) strains. These strains displayed an identity of 97.62–98.86 and 98.41–99.17 percent in comparison to the Russian strains, respectively (Figure 4). It is noteworthy that the number of differences between the Russian Far East isolates was occasionally higher than that between the isolates and foreign ones, which suggests the possibility of multiple transboundary introgressions. The VP1 gene sequence pairwise comparisons confirm this observation (Supplementary Figure S3).

### 3.4. Vaccine Matching

To identify the most optimal and effective vaccine strains, an analysis of the antigenic characteristics of the isolated FMDV type A strains was performed. A cross-microneutralization reaction was conducted to assess the antigenic compatibility of FMDV type A isolates from the Far East and North Caucasus regions of the Russian Federation with the vaccine strain A_22_. The antigenic matching of FMDV type A isolates from the Far East and North Caucasus regions of the Russian Federation with vaccine strains A_22_/550, A/Iran/97, A/Turkey/06, and A/Kyrgyzstan/07 was conducted using reference serum samples from cattle immunized with monovalent vaccines. The results of these matching studies are presented in Table 4.

As illustrated by the data presented in Table 4, the isolates from outbreaks in the North Caucasus demonstrated antigenic variations from the type A vaccine strains utilized for comparison: A_22_/550, A_22_/Iraq/64, A/Iran/97, A/Turkey/06, and A/Kyrgyzstan/07 (r_1_ < 0.3). A similar antigenic dissimilarity was observed between the Far Eastern isolates and the vaccine strains of type A, which included the strains A_22_/550, A_22_/Iraq/64, A/Iran/97, and A/Kyrgyzstan/07 (r_1_ < 0.3). However, the FMDV type A isolates No. 2155, 2156, 2175, and 2177, isolated in 2013 in the Zabaykalsky Krai and Amur Oblast, demonstrated antigenic relatedness (r_1_ 0.35–0.48) to the A/Turkey/06 strain.

The obtained results in Table 4 indicated the presence of close antigenic relatedness between the production strain A/2155/Zabaikalsky/2013 and the FMDV isolates A/2177/Amursky/2013, A/2203/Zabaikalsky/2014, A/2225/Zabaikalsky/2014, and A/2233/Zabaikalsky/2014 (r_1_ 0.46–0.74). Moreover, a close antigenic relatedness between the production strain A/2166/Krasnodarsky/2013 and the isolate A/2171/Kabardino-Balkarian/2013 (r_1_ = 0.69) was observed.

## 4. Discussion

The current FMD prevention and control policy in the Russian Federation is based on three main strategies [31]. The first strategy is the implementation of export and import controls in accordance with the recommendations set forth in the World Organization for Animal Health (WOAH) Terrestrial Animal Health Code. The second strategy is the implementation of a zoning plan for the country’s territory. The third strategy is the application of a preventive vaccination program for cattle and small ruminants in regions bordering affected countries. In order to vaccinate animals against FMD, the Russian Federation successfully employs the use of domestically produced inactivated vaccines with high protection profiles. These vaccines contain FMDV antigens of the A, O, and Asia-1 serotypes, depending on the epidemiological context [32,33].

Russia is not endemic to foot-and-mouth disease [34], yet the potential for a spillover from neighboring countries in Asia persists. This is due to the constant threat of the disease being transmitted from affected regions through cattle or wild animals [35]. Outbreaks of foot-and-mouth disease caused by incursions from outside have been reported in Russia since 1995, with subsequent outbreaks occurring in 2000, 2004–2006, and 2010–2014 [36,37,38]. It is noteworthy that prior to 2013, waves of FMD outbreaks were attributed to type O and Asia 1 lineages, which exhibited similarities at the VP1 target to FMD from China, Kazakhstan, and Mongolia [39,40,41].

The 2013–2014 outbreaks of FMD resulted in the emergence of a novel epidemiological situation in geographically distant regions, including the Far East and southern Russia. A considerable number of outbreaks were recorded in Zabaykalsky Krai (nine outbreaks) and the Amur Oblast (six outbreaks), as well as, for the first time in many decades, in the Karachaevo-Cherkesian Republic (two outbreaks), Krasnodarsky Krai (three outbreaks), and the Kabardino-Balkarian Republic (one outbreak) (Figure 1).

In this study, we employed full-genome sequencing and phylogenetic reconstruction across the entire genome and the VP1 gene locus to conduct a comprehensive analysis of the molecular epidemiology of FMD in Russia during the 2013–2014 period. Representative isolates were selected based on information from the FMD reference laboratory and data from the literature regarding outbreaks that occurred in geographically proximate regions. The phylogenetic analysis demonstrated that the sequenced strains were representative of two distinct genetic lineages of serotype A: SEA97 in the Far East and Iran-05 in the North Caucasus. The topology of the phylogenetic trees obtained for the complete genome and the VP1 gene is similar, indicating both the reliability of the analysis and the unlikelihood of earlier recombination events in the isolates under study (Figure 2 and Figure 3).

All outbreaks in the Far East in 2013 were caused by a virus belonging to the genetic lineage A/Sea-97 (A/Southeast Asia-97). The Sea-97 lineage is divided into five major groups, designated G1-G5, of which the isolates studied belong to the G2 group along with isolates from China, Thailand, and Vietnam [42,43]. This group is endemic to the countries of Southeast Asia: Vietnam, Thailand, Malaysia, Laos, and Cambodia [42]. Literature analysis indicates that since 2013, this virus has gone beyond its natural range and spread to China, Kazakhstan, and Mongolia, from where it entered Russia. The high level of genetic similarity between the Russian and Chinese isolates (Figure 2, Figure 3 and Figure 4) supports the relatedness of these strains and, taking into account the published reports of the Sea-97 lineage in China in as early as 2009 (https://www.wrlfmd.org/country-reports/country-reports-2009, accessed on 19 December 2024), suggests the most likely route of transmission from south to north [44,45]. The observed expansion events indicate a new twist in the ever-changing epidemiology of FMD in East Asia and increase the risk of further range expansion to more distant countries, including FMD-free countries. A/ASIA/Sea-97 was the most common topotype among serotype A viruses in sporadic or endemic outbreaks in Southeast, Central, and East Asia between 2009 and 2020.

By contrast, outbreaks reported in the southern regions of Russia were attributed to the Iran-05 lineage of the Asia topotype, which was initially identified in Iran in 2003 during the 2005–2006 epidemic. It subsequently disseminated throughout the Middle East, becoming established in this region [46]. Since the onset of the Middle East epidemic, the lineage has undergone evolutionary changes, giving rise to numerous sublineages within the genetic lineage [47]. Many of the sublineages were relatively short-lived, disappearing shortly after their initial appearance. However, some were able to persist and dominate for a considerable period of time [42,48]. The available genetic and epidemiological data since 2011 indicate that the SIS-10 sublineage of the A/Iran-05 lineage, to which the Russian isolates belong (see Figure 2 and Figure 3), has been the dominant strain across Turkey [46,49]. According to the reports from the World Reference Laboratory for Foot-and-Mouth Disease (WRLFMD, in Pirbright, UK), isolates of the genetic sublineage A/ASIA/Iran-05/SIS-10 served as the causative agents of FMD outbreaks in Middle Eastern countries between 2011 and 2013. This raises the question of the origin of these spillover events. While the introduction of FMD from Turkey into the North Caucasus is unlikely, the most probable scenario is that the A/Iran-05 SIS10 sublineage spread across the Transcaucasian countries, subsequently spilling over into the North Caucasus.

The incursions of two different lineages necessitated a re-examination of the control and eradication policies that were in place at that time. The isolates under study exhibited antigenic differences from the type A vaccine strains used for comparison, including A_22_/550, A_22_/Iraq/64, A/Iran/97, A/Turkey/06, and A/Kyrgyzstan/07 (r_1_ < 0.3). These findings are consistent with the results of the phylogenetic and antigenic relatedness study conducted by Mahapatra and colleagues [28], which indicated that the virus isolates circulating in the Middle East in 2012–2013 belonged to the SIS-10 sublineage. The A-Iran-05 genetic lineage exhibited antigenic drift and a low antigenic match with the vaccine strains A_22_/IRQ/64 and, in particular, A/Turkey/06, which have been the predominant strains utilized for FMD control in this region since 2006 [28].

Isolates from the Zabaikalsky Krai and Amur Oblast in 2013-2014, belonging to the “Southeast Asia-Sea-97” genetic lineage, “Asia” topotype also exhibited antigenic divergence from strains A_22_/550, A_22_/Iraq/64, A/Iran/97, and A/Kyrgyzstan/07 (r_1_ < 0.3). Moreover, the FMDV isolates A-2155, A-2156, A-2175, and A-2177, registered in 2013 in the Zabaikalsky Krai and Amur Oblast, showed antigenic relatedness (r_1_ 0.35–0.48) to the A/Turkey/06 strain. As a consequence of the identified antigenic dissimilarities observed between the isolates and the available vaccine strains, the newly obtained strains of FMDV A/2155/Zabaykalsky/2013 and A/2166/Krasnodarsky/2013 were officially registered and deposited in the All-Russian State Collection of Exotic Types of Foot-and-Mouth Disease Virus and Other Animal Pathogens of the Federal Center for Animal Health “ARRIAH”. Also, these strains were recommended for inclusion in the trivalent vaccine in the FMD-free regions of the Russian Federation.

In conclusion, the genetic analysis of FMDV isolates from various regions of the Russian Federation in 2013–2014 revealed that these isolates belonged to two different lineages: the SEA-97 and Iran-05. The present study underscores the necessity for a more rigorous FMD surveillance program in the highlighted regions, the importance of developing prompt intervention strategies, continuous surveillance, and vaccine strain selection for effective management and control of FMDV.

## Figures and Tables

**Table 1:** FMDV serotype A isolates caused FMD outbreaks in the Russian Federation in 2013–2014.

Year	Region	OIE Notification Date	Topotype	Genetic Lineage	Isolate Name
2013	Zabaykalsky Krai	18.03.13	ASIA	Sea-97	A-2182/Zabaykalsky/2013
2013	Amur Oblast	28.06.13 08.07.13	ASIA	Sea-97	A-2170/Amursky/2013
2013	Krasnodar Krai	17.06.13	ASIA	Iran-05	A-2169/Krasnodar/2013
2013	Karachay-Cherkess Republic	07.06.13 10.07.13	ASIA	Iran-05	A-2167/Karachay-Cherkessia/2013
2013	Kabardino-Balkarian Republic	17.07.13	ASIA	Iran-05	A-2171/Kabardino-Balkaria/2013
2014	Zabaykalsky Krai	25.01.14 11.09.14	ASIA	Sea-97	A-2203/Zabaykalsky/2014 A-2225/Zabaykalsky/2014

**Table 2:** Titration results of the FMD virus type A isolates.

FMDV Isolate	Titration/ TCD_50_/mL	Genetic Lineage
A-2167/Karachay-Cherkessia/2013	5.5	A-Iran-05
A-2169/Krasnodar/2013	5.25	A-Iran-05
A-2170/Amursky/2013	5.21	A/ASIA/SEA-97
A-2171/Kabardino-Balkaria/2013	5.82	A-Iran-05
A-2182/Zabaykalsky/2013	4.57	A/ASIA/SEA-97
A-2203/Zabaykalsky/2014	5.81	A/ASIA/SEA-97
A-2225/Zabaykalsky/2014	5.13	A/ASIA/SEA-97

**Table 3:** Sequencing and de novo assembly results of FMDV isolates in this study.

FMDV Isolate	Number of Reads, Millions of Read Pairs	Viral Median Genome Coverage, × (% of Total Reads)	Genome Length, bp	GenBank acc.	SRA acc.
A-2167/Karachay-Cherkessia/2013	10.39	3661 (2.53%)	8126	PQ474240	SRR31444037
A-2169/Krasnodar/2013	15.15	9721 (3.83%)	8181	PQ474239	SRR31444036
A-2170/Amursky/2013	9.06	6115 (6.85%)	8259	PQ474238	SRR31444035
A-2171/Kabardino-Balkaria/2013	17.07	45,142 (10.85%)	8232	PQ474237	SRR31444034
A-2182/Zabaykalsky/2013	13.57	34,363 (10.21%)	8275	PQ474236	SRR31444033
A-2203/Zabaykalsky/2014	23.02	38,212 (6.51%)	8178	PQ474235	SRR31444032
A-2225/Zabaykalsky/2014	15.82	21,256 (7.47%)	8129	PQ474234	SRR31444031

**Table 4:** Results of the antigenic matching (r_1_ *) of FMDV type A isolates.

FMDV Isolate	A_22_/550	A_22_/Iraq/64	A/Iran/97	A/2029/ Turkey/06	A/2045/ Kyrgyzstan/07	A/2155/ Zabaykalsky/13	A/2166/ Krasnodarsky/13
A/2155/ Zabaykalsky/13	0.13	0.27	0.036	0.37	0.14	n/a **	n/a
A/2156/ Zabaykalsky/13	0.22	0.4	0.05	0.35	0.12	n/a	n/a
A/2166/ Krasnodar/13	0.015	0.02	0.025	0.13	0.03	n/a	n/a
A/2167/ Karachay-Cherkessia/13	0	0	0.02	0.12	0.009	n/a	n/a
A/2169/ Krasnodar/13	0.01	0	0.02	0.17	0.01	n/a	n/a
A/2170/ Amur/13	0.04	0.3	0.03	0.24	0.02	n/a	n/a
A/2171/ Kabardino-Balkaria/13	0.018	0.1	n/a	0.19	0.03	0.05	0.69
A/2175/ Amursky/13	0.04	0.38	0.01	0.38	0.02	n/a	n/a
A/2177/ Amursky/13	0.29	n/a	0.03	0.48	n/a	0.74	n/a
A/2203/ Zabaykalsky/14	n/a	n/a	n/a	n/a	n/a	0.48	0.05
A/2225/ Zabaykalsky/14	0.17	0.04	0.7	0.28	0.15	0.64	n/a

* r_1_ ≥ 0.3 indicates a close relationship between the field isolate and the vaccine strain. A potent vaccine containing the vaccine strain is likely to confer protection; r_1_ < 0.3 indicates that the field isolate is so different from the vaccine strain that the vaccine is unlikely to protect; ** n/a—not analyzed.

## Data Availability

All genomic data related to the project, including sample description and sequencing reads were deposited to the NCBI database under the BioProject PRJNA1189077. The assembled and annotated viral genome sequences were deposited to GenBank under accession numbers PQ474234-PQ474240.

## Supplementary Materials

The following supporting information can be downloaded at: https://www.mdpi.com/article/10.3390/v17010008/s1, Figure S1: The pairwise comparisons of the VP1 gene sequences of serotype A FMDV; Table S1: The dataset of 52 genomes utilized for the reconstruction of the whole genome phylogenetic tree; Table S2: The dataset of 79 VP1 gene sequences utilized for the reconstruction of the VP1 phylogenetic tree.
