# Supplementary material for: The Presence of Two Distinct Lineages of the Foot-And-Mouth Disease Virus Type A in Russia in 2013–2014 Has Significant Implications for the Epidemiology of the Virus in the Region
Source: Viruses. 2024 Dec 25;17(1):8. doi: 10.3390/v17010008 (PMC11769220; doi:10.3390/v17010008)
Supplement: Supplementary file 1 [file viruses-17-00008-s001.zip › viruses-3365076-supplementary.pdf]

SUPPLEMENTARY MATERIALS

The presence of two distinct lineages of foot-and-mouth disease virus type A in Russia in 2013-2014 has significant implications for the epidemiology of the virus in the region.

Victor V. Nikiforov[1], Sergey A. Noskov[2], Alexander V. Sprygin[1], Muhamad Abed Alhussein[1], Anastasia S. Krylova[2], Taisia V. Erofeeva[2], Svetlana N. Fomina[1], Svetlana R. Kremenchugskaya[1], Fedor I. Korennoy[1], Maxim V. Patrushev[2], Ilya A. Chvala[1], Tamara K. Mayorova[1] and Stepan V. Toshchakov[2,*]

[1] Federal Center for Animal Health FGBI ARRIAH, Vladimir, Russia; arriah@fsvps.gov.ru
[2] National Research Center "Kurchatov Institute", Moscow, Russia; nrcki@nrcki.ru
* Correspondence: toschakov_sv@rrcki.ru; stepan.toshchakov@gmail.com

SUPPLEMENTARY TABLES

Supplementary Table 1. The dataset of 52 genomes utilized for the reconstruction of the whole genome phylogenetic tree.

ID	Isolate	Origin	Collection_ year	Length	GC(%)
**AY593755**	A15/Bangkok/TAI/60	Thailand	1960	8192	54.03
**AY593761**	A21/Lumbwa/KEN/3/64	Kenya	1964	8161	54.32
**AY593763**	A22/IRQ/24/1964	Iraq	1964	8204	52.94
**AY593766**	A23/Kenya/6/65	Kenya	1965	8202	54.26
**AY593768**	A24/Cruzeiro/BRA/55	Brazil	1955	8156	53.76
**AY593780**	A5/Allier/FRA/60	France	1960	8199	54.07
**EF494486**	A/TUR/2005	Turkey	2005	7649	53.41
**EF494487**	A/PAK/1/2006	Pakistan	2006	7656	53.3
**FMD-A-2167**	A-2167/Karachay-Cherkessia/2013	Karachay-Cherkess_Republic	2013	8129	53.68
**FMD-A-2169**	A-2169/Krasnodar/2013	Krasnodar_Krai	2013	8178	53.72
**FMD-A-2170**	A-2170/Amursky/2013	Amur_Oblast	2013	8275	54.16
**FMD-A-2171**	A-2171/Kabardino-Balkaria/2013	Kabardino-Balkar_Republic	2013	8232	53.72
**FMD-A-2182**	A-2182/Zabaykalsky/2013	Zabaykalsky_Krai	2013	8259	54.04
**FMD-A-2203**	A-2203/Zabaykalsky/2014	Zabaykalsky_Krai	2014	8181	53.83
**FMD-A-2225**	A-2225/Zabaykalsky/2014	Zabaykalsky_Krai	2014	8126	53.8

Supplementary Table 1. The dataset of 52 genomes utilized for the reconstruction of the whole genome phylogenetic tree *(continued)*.

ID	Isolate	Origin	Collection_ year	Length	GC(%)
**GQ406247**	A/VN/09/2009	Viet Nam	2009	8207	53.59
**GQ406248**	A/VN/02/2009	Viet Nam	2009	8210	53.45
**GQ406249**	A/VN/03/2009	Viet Nam	2009	8206	53.58
**GQ406251**	A/VN/16/2009	Viet Nam	2009	8207	53.61
**GQ406252**	A/VN/20/2009	Viet Nam	2009	8215	53.45
**HM854022**	A/IND/17/1977	India	1977	8207	53.8
**HM854024**	A/IND/17/1982	India	1982	8158	53.16
**HQ632773**	A/MAY/3/2007	Malaysia: Kuala Selangor, Selangor	2007	8192	53.48
**HQ832576**	A/IND/21/1990	India: Assam	1990	8187	53.89
**HQ832585**	A/IND/26/2006	India: Gujarat	2005	8183	53.29
**HQ832587**	A/IND/50/2006	India: Karnataka	2005	8190	53.81
**JF749843**	A/EGY/2006	Egypt	2006	8195	53.78
**JF749848**	A/TUR/004/2003	Turkey	2003	7826	53.13
**JN006722**	A/SIN/PAK/L4/2008	Pakistan	2008	7635	53.16
**JN099693**	A/IRQ/4239/09	Iraq: Abu-gareb, Baghdad	2009	7415	53.24
**JN099699**	A/IRQ/4255/09	Iraq: Maakal, Basrah	2009	7531	53.35
**KC588943**	A/KOR/Pocheon-001/2010	South Korea	2010	8196	53.34
**KJ608371**	A/VN/T11D/2013	Viet Nam	2013	8229	54.2
**KJ754939**	A/BAN/GA_Sa-197/2013	Bangladesh	2013	8220	53.13
**KM268896**	A/TUR/11/2013	Turkey: Nazirlar, Mengen, Bolu, Marmara	2013	8211	53.42
**KP940474**	A/EGY/El-Fayoum/2014	Egypt	2014	7281	53.59
**KT968663**	A/CHA/HY/2013	China	2013	8103	54.08
**KY322676**	A/MAY/12/2013	Malaysia: Seberang Jertih, Besut, Terengganu	2013	8196	53.83

Supplementary Table 1. The dataset of 52 genomes utilized for the reconstruction of the whole genome phylogenetic tree *(continued)*.

ID	Isolate	Origin	Collection_ year	Length	GC(%)
**KY404934**	A/ARG/01L/2001	Argentina	2001	8211	53.73
**LC564900**	A/TAI/25/2009	Thailand:Udon Thani	2009	7625	53.18
**LC564904**	A/TAI/73/2010	Thailand:Uttaradit	2010	7625	53.94
**LC564908**	A/TAI/1/2012	Thailand:Pattani	2012	7625	53.52
**LC564917**	A/TAI/46-1/2015	Thailand:Songkhla	2015	7632	53.64
**MH053305**	A/EGY/1/72	Egypt: Alexandria	1972	8209	53.25
**MK341544**	A/Arg/01-CapLc/2001	Argentina	2001	8211	53.7
**MZ493234**	A/PAK/C8-Clone-03/2018	Pakistan: Punjab, Chakwal	2018	7743	53.55
**OK205896**	A/VIT/8506/2014	Viet Nam: Kon Tum	2014	6999	53.72
**OK205897**	A/VIT/16451DLBPP01/2015	Viet Nam: Dak Lak	2015	6999	53.56
**OK205898**	A/VIT/16478XL15/2013	Viet Nam: Ha Tinh	2013	6999	53.61
**OK205899**	A/VIT/16705/2015	Viet Nam: Phu Yen	2015	6999	53.62
**OK205904**	A/VIT/NCVDF1422/2014	Viet Nam: Bac Kan	2014	6999	53.72
**OK318503**	A/VIT/DT-P73-4/2019	Viet Nam: Dong Thap	2019	8177	53.81

Supplementary Table 2. The dataset of 79 VP1 gene sequences utilized for the reconstruction of the VP1 phylogenetic tree.

ID	Isolate	Origin	Collection year	Length, bp	GC (%)
**AF390646**	A/IND/40/2000*	India: Karnataka	1999	639	57.43
**AJ306219**	A/Alem/ARG/81	Argentina:Buenos Aires	1981	639	56.18
**AY593755**	A15/Bangkok/TAI/60	Thailand	1960	639	56.65
**AY593761**	A21/Lumbwa/KEN/3/64	Kenya	1964	639	56.49
**AY593763**	A22/IRQ/24/1964	Iraq	1964	639	55.71
**AY593766**	A23/Kenya/6/65	Kenya	1965	639	58.53
**AY593768**	A24/Cruzeiro/BRA/55	Brazil	1955	639	55.87

Supplementary Table 2. The dataset of 79 VP1 gene sequences utilized for the reconstruction of the VP1 phylogenetic tree *(continued)*.

ID	Isolate	Origin	Collection year	Length, bp	GC (%)
**AY593780**	A5/Allier/FRA/60	France	1960	639	56.49
**EF208756**	A/EGY/1/72	Egypt: Alexandria	1972	639	57.43
**EF208769**	A/IRN/1/2005	Iran: Ghalch-Sadri, Qom, Qom Province	2005	639	57.12
**EF208770**	A/IRN/2/87	Iran: Mardabad, Kardaj, Tehran	1987	639	58.69
**EF208771**	A/IRN/1/96	Iran: Zarnan, Shahriar, Tehran	1996	639	59
**EF208772**	A/IRN/22/99	Azerbaijan: Tabriz, East Province, Iran	1999	639	57.9
**EF208777**	A/TAI/118/87*	Thailand: Sara Buri	1987	639	58.37
**EF208778**	A/TAI/2/97	Thailand	1997	639	58.69
**EF494486**	A/TUR/2005	Turkey	2005	639	57.12
**EF494487**	A/PAK/1/2006	Pakistan	2006	639	57.28
**EU553852**	A11/Germany/AGB/29	Germany	1929	639	56.03
**FJ755007**	A/AFG/6/2007	Afghanistan	2007	639	57.9
**FJ755010**	A/BAR/6/2008	Bahrain	2008	639	56.81
**FJ755133**	A/TUR/1/2008	Turkey	2008	639	56.96
**FJ755155**	A/TUR/33/2008	Turkey	2008	639	56.96
**FMD-A-2167**	A-2167/Karachay-Cherkessia/2013	Karachay-Cherkess_Republic	2013	639	57.12
**FMD-A-2169**	A-2169/Krasnodar/2013	Krasnodar_Krai	2013	639	57.12
**FMD-A-2170**	A-2170/Amursky/2013	Amur_Oblast	2013	639	59
**FMD-A-2171**	A-2171/Kabardino-Balkaria/2013	Kabardino-Balkar_Republic	2013	639	56.49
**FMD-A-2182**	A-2182/Zabaykalsky/2013	Zabaykalsky_Krai	2013	639	58.84
**FMD-A-2203**	A-2203/Zabaykalsky/2014	Zabaykalsky_Krai	2014	639	58.69
**FMD-A-2225**	A-2225/Zabaykalsky/2014	Zabaykalsky_Krai	2014	639	58.69

Supplementary Table 2. The dataset of 79 VP1 gene sequences utilized for the reconstruction of the VP1 phylogenetic tree *(continued)*.

ID	Isolate	Origin	Collection year	Length, bp	GC (%)
**GQ406247**	A/VN/09/2009	Viet Nam	2009	639	58.37
**GQ406248**	A/VN/02/2009	Viet Nam	2009	639	58.22
**GQ406249**	A/VN/03/2009	Viet Nam	2009	639	58.22
**GQ406251**	A/VN/16/2009	Viet Nam	2009	639	58.06
**GQ406252**	A/VN/20/2009	Viet Nam	2009	639	58.37
**GU566064**	A/SUD/3/77	Sudan	1977	639	56.96
**HM854022**	A/IND/17/1977	India	1977	639	58.69
**HM854024**	A/IND/17/1982	India	1982	639	57.59
**HQ116312**	A/TAI/7/2003	Thailand	2003	639	58.53
**HQ632773**	A/MAY/3/2007	Malaysia: Kuala Selangor, Selangor	2007	639	59.15
**HQ832576**	A/IND/21/1990	India: Assam	1990	639	59
**HQ832585**	A/IND/26/2006	India: Gujarat	2005	639	57.28
**HQ832587**	A/IND/50/2006	India: Karnataka	2005	639	56.81
**JF749843**	A/EGY/2006	Egypt	2006	639	55.71
**JF749848**	A/TUR/004/2003	Turkey	2003	639	57.59
**JN006722**	A/SIN/PAK/L4/2008	Pakistan	2008	639	57.75
**JN099693**	A/IRQ/4239/09	Iraq: Abu-gareb, Baghdad	2009	639	56.96
**JN099699**	A/IRQ/4255/09	Iraq: Maakal, Basrah	2009	639	56.96
**KC588943**	A/KOR/Pocheon-001/2010	South Korea	2010	639	58.37
**KF561698**	A/GHA/16/73	Ghana	1973	639	55.71
**KF561699**	A/KEN/42/66	Kenya	1966	639	58.37
**KF561704**	A/NGR/2/73	Niger: Niamey	1973	639	56.65
**KF561705**	A/UGA/13/66	Uganda	1966	639	57.12

Supplementary Table 2. The dataset of 79 VP1 gene sequences utilized for the reconstruction of the VP1 phylogenetic tree *(continued)*.

ID	Isolate	Origin	Collection year	Length, bp	GC (%)
**KJ608371**	A/VN/T11D/2013	Viet Nam	2013	639	58.84
**KJ754939**	A/BAN/GA_Sa-197/2013	Bangladesh	2013	639	55.56
**KM268896**	A/TUR/11/2013	Turkey: Nazirlar, Mengen, Bolu, Marmara	2013	639	57.43
**KP940474**	A/EGY/El-Fayoum/2014	Egypt	2014	639	58.53
**KT968663**	A/CHA/HY/2013	China	2013	639	59.15
**KY091290**	A/IRN/9/2010	Iran: M Foladi, Esfahan, Esfahan	2010	639	57.75
**KY091291**	A/IRN/78/2009	Iran: Shiraz, Fars	2009	639	57.59
**KY091293**	A/AFG/10/2010	Afghanistan: Qala-I-Reg, 9th, Hirat	2010	639	59
**KY091294**	A/TUR/3/2010	Turkey: Gulyazi, Kixiloren, Afyon	2010	639	58.84
**KY091295**	A/IRN/9/2011	Iran: Qazvin	2011	639	57.43
**KY091296**	A/IRN/125/2010	Iran: Sistan and Baluchistan	2010	639	58.06
**KY091297**	A/IRN/15/2012	Iran: Zahedan, Sistan and Baluchestan	2012	639	57.75
**KY322676**	A/MAY/12/2013	Malaysia: Seberang Jertih, Besut, Terengganu	2013	639	58.06
**KY404934**	A/ARG/01L/2001	Argentina	2001	639	55.09
**LC564900**	A/TAI/25/2009	Thailand:Udon Thani	2009	639	57.75
**LC564904**	A/TAI/73/2010	Thailand:Uttaradit	2010	639	59
**LC564908**	A/TAI/1/2012	Thailand:Pattani	2012	639	58.53
**LC564917**	A/TAI/46-1/2015	Thailand:Songkhla	2015	639	59
**MH053305**	A/EGY/1/72	Egypt: Alexandria	1972	639	57.43

Supplementary Table 2. The dataset of 79 VP1 gene sequences utilized for the reconstruction of the VP1 phylogenetic tree *(continued)*.

ID	Isolate	Origin	Collection year	Length, bp	GC (%)
**MK341544**	A/Arg/01-CapLc/2001	Argentina	2001	639	55.4
**MZ493234**	A/PAK/C8-Clone-03/2018	Pakistan: Punjab, Chakwal	2018	639	56.49
**OK205896**	A/VIT/8506/2014	Viet Nam: Kon Tum	2014	639	57.12
**OK205897**	A/VIT/16451DLBPP01/2015	Viet Nam: Dak Lak	2015	639	57.43
**OK205898**	A/VIT/16478XL15/2013	Viet Nam: Ha Tinh	2013	639	57.12
**OK205899**	A/VIT/16705/2015	Viet Nam: Phu Yen	2015	639	56.65
**OK205904**	A/VIT/NCVDF1422/2014	Viet Nam: Bac Kan	2014	639	57.12
**OK318503**	A/VIT/DT-P73-4/2019	Viet Nam: Dong Thap	2019	639	56.18

SUPPLEMENTARY FIGURES

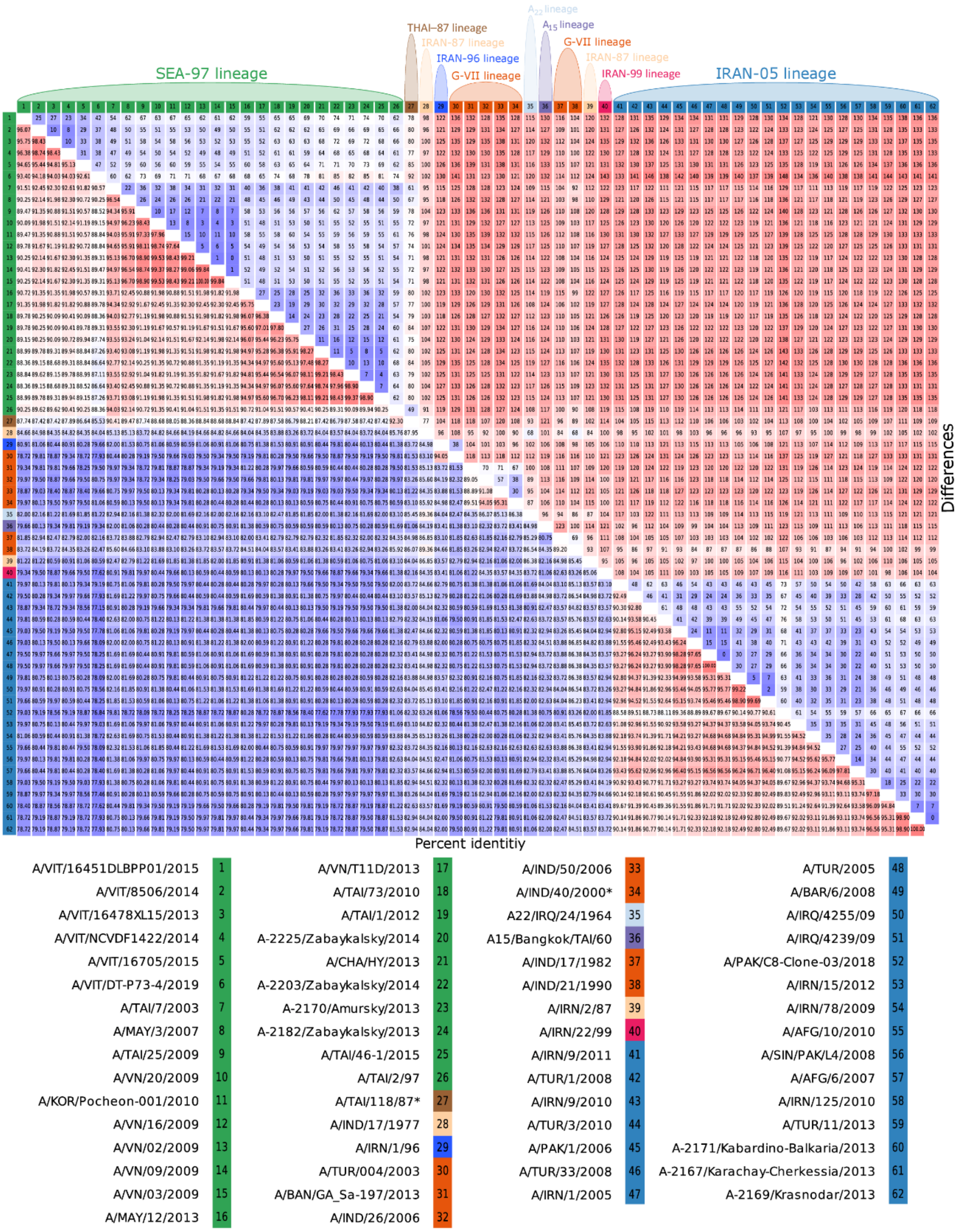

Supplementary Figure 1. The pairwise comparisons of the VP1 gene sequences of serotype A FMDV. The percentage of identical nucleotides is displayed in the bottom left half of the heatmap. The top right half depicts the number of differences between the two sequences, including both gaps and single nucleotide polymorphisms (SNPs).
